# Tribological Properties of Selected Ionic Liquids in Lubricated Friction Nodes

**DOI:** 10.3390/ma18010018

**Published:** 2024-12-24

**Authors:** Monika Madej, Joanna Kowalczyk, Marcin Kowalski, Paweł Grabowski, Jacek Wernik

**Affiliations:** 1 Faculty of Mechatronics and Mechanical Engineering, Kielce University of Technology, 25-314 Kielce, Poland; mmadej@tu.kielce.pl; 2 Faculty of Civil Engineering, Mechanics and Petrochemistry, Warsaw University of Technology, 09-400 Płock, Poland; marcin.kowalski2@pw.edu.pl (M.K.); pawel.grabowski@pw.edu.pl (P.G.); jacek.wernik@pw.edu.pl (J.W.)

**Keywords:** ionic liquid, wear, friction

## Abstract

This article compares the rheological and tribological properties of three ionic liquids: Tributyl(methyl)phosphonium dimethyl phosphate 97%—MFCD, 1-Butyl-3-methylimidazolium hexafluorophosphate 97%—BMIMPF6, and 1-Butyl-3-methylimidazolium tetrafluoroborate 98%—BMIMBF4. Their density and kinematic viscosity at 20 °C and 40 °C were investigated, and tribological tests were carried out at the same temperatures with ball-on-disc contact. The test materials were made of 100Cr6 steel. A scanning electron microscope was used to image the wear tracks, while an EDS analyzer was employed to determine the chemical composition at the points of wear on the samples. A confocal microscope was used to analyze the geometric structure of the samples before and after the tribological tests. The results of the tests indicated that an increase in temperature reduced the dynamic viscosity of all the ionic liquids tested. At the same time, an increase in the MFCD and BMIMBF4 ionic liquid density and a decrease in the density of the BMIMPF6 ionic liquid were observed. The BMIMPF6 ionic liquid used for this study provided the lowest value of linear wear at both temperatures, ambient and 40 °C. However, for the BMIMBF4 ionic liquid, significant wear was observed for the tested discs and balls, with corrosive pitting on their surfaces.

## 1. Introduction

Nearly half of all mechanical component failures can be attributed to inadequate lubrication in mechanical assemblies [1]. Friction poses a significant global challenge, as approximately 30% of the world’s energy production is consumed in overcoming frictional forces [2], contributing to increased carbon dioxide emissions [3]. Lubricants offer a promising solution to reduce wear and friction [1]. To mitigate the adverse effects of friction, various techniques have been explored, including surface modification methods like physical vapor deposition (PVD) and chemical vapor deposition (CVD) [4], as well as laser-based [5,6] and electrochemical methods [7]. Additionally, various additives, such as graphene, graphene oxide [8], and ionic liquids, have been introduced to enhance lubrication properties. Given the increasing environmental concerns, regulations are now promoting the use of more eco-friendly lubricants [9]. Ionic liquids, often referred to as “green solvents”, offer a promising solution in this regard [10,11,12].

The use of ionic liquids in tribology, either as lubricants [13,14,15,16] or lubricant additives, is a relatively recent development, first discovered in 2001 [13,14]. Ionic liquids possess several properties that make them attractive for tribological applications, including negligible vapor pressure [13,14,17,18], high thermal stability [13,14,17,18,19,20,21,22], non-combustibility [13,14,17,18,19,22], non-volatility [13,14], and electrochemical properties [13,14,23]. However, their high polar structure limits their solubility in oils, which can hinder their practical use. This limitation can be partially addressed by modifying their structure, such as elongating their alkyl chains [13,14]. Ionic liquids (ILs) undergo tribochemical reactions with interacting metal surfaces, forming anti-wear tribofilms that enhance the lubricant’s low-friction and anti-wear properties. Furthermore, ILs are known as designer fluids because by modifying the cation–anion combination [24,25], it is possible to tailor the properties of ionic liquids, such as their solubility and miscibility with solvents [24].

In tribology applications, only a few specific structures can be used as lubricants [13,25,26] and lubricant additives [13,25] due to their incorporation of elements that enhance tribological properties. ILs containing fluorine and phosphorus within their molecular structures have exhibited anti-wear and anti-corrosion performance. Phosphorus forms a phosphate film on the sliding surface through tribochemical reactions. The formation of this phosphate boundary layer prevents the formation of iron halide and consequently increases wear resistance [27].

The influence of phosphorus in ionic liquids on improving tribological properties was investigated by Jiménez et al. [28], who used an ionic liquid based on the 1-N-alkyl-3-methylimidazolium cation with tetrafluoroborate and hexafluorophosphate anions as pure lubricants and as lubricant additives. Experiments were conducted at room temperature and 100 °C. The results demonstrated a significant enhancement in lubrication performance (reduced friction and wear) when 1% of the ionic liquid was added to the base oil compared to the pure base oil. In another study, Jiménez et al. [29] compared the lubricating properties of imidazolium-based ionic liquids at various temperatures. 1-Hexyl/octyl-3-methylimidazolium tetrafluoroborate exhibited superior thermal stability and lubrication performance compared to mineral and synthetic oils under extreme temperatures (−30 °C, 100 °C, and 200 °C).

Madej et al. [30] assessed the influence of a-C:H and a-C:H:W coatings on corrosion and tribological properties. Tribological tests were conducted under dry friction conditions and lubricated conditions using 1-butyl-3-methylimidazolium tetrafluoroborate (BMIBF4) as the lubricant. The ionic liquid contributed to reduced friction coefficients and linear wear in the tested tribological systems.

Totolin and his team [31] compared the tribological performance of various ionic liquids. They investigated alkylborate ionic liquids containing imidazole and phosphonium phosphate. The results indicated that phosphate tribofilms formed from phosphonium phosphate-based ionic liquids exhibited superior tribological properties compared to fluorine-containing tribofilms derived from halogenated ionic liquids.

Wang et al. [32] tested alkyl imidazolium hexafluorophosphate-type ionic liquids as a lubricant in steel–steel contact. Tribological tests were performed on the Optimol SRV oscillating friction and wear tester under ambient conditions and on a CZM vacuum friction tester under 1 × 10^−3^ Pa pressure. The synthetic ionic liquids exhibited excellent tribological performance (low friction coefficient and wear), superior to conventional lubricants in liquid paraffin containing 2 wt.% zinc dialkyldithiophosphate (ZDDP). In addition to wear, the researchers observed the presence of phosphorus at the friction point on the steel surfaces. Moreover, they noted the occurrence of a tribochemical reaction and the formation of FePO_4_ and FeF_2_ from the ionic liquid used in the tests.

This study aimed to compare the tribological properties of three selected ionic liquids: Tributyl(methyl)phosphonium dimethyl phosphate, 1-Butyl-3-methylimidazolium hexafluorophosphate 97%, and 1-Butyl-3-methylimidazolium tetrafluoroborate. The ionic liquids used in this study contain phosphorus and/or fluorine. Experiments were conducted at two temperatures, ambient and 40 °C. However, the primary objective of this study, which directly stemmed from the research conducted, was to determine the influence of phosphorus and fluorine from the ionic liquids on the wear of 100Cr6 steel.

## 2. Materials and Methods

Three ionic liquids were used in this study: Tributyl(methyl)phosphonium dimethyl phosphate 97%—MFCD, 1-Butyl-3-methylimidazolium hexafluorophosphate 97%—BMIMPF6, a widely used commercial ionic liquid which is viscous, colorless, hygroscopic, and hydrophobic, with poor water solubility due to the hydrophobicity of the hexafluorophosphate anion [10], and 1-Butyl-3-methylimidazolium tetrafluoroborate 98%—BMIMBF4. All ionic liquids were commercially sourced from Sigma-Aldrich (USA). Table 1 and Table 2 provide a summary of their key properties [33,34].

Density was determined using a Mettler-Toledo automatic densitometer (Nänikon, Switzerland) with a temperature accuracy of 0.2 °C and a repeatability of 0.0005 g/cm^3^. Each sample was measured three times at the specified temperature.

The results of the experiments are presented in Section 3.1.

Dynamic viscosity was measured using an IKA ROTAVISC me-vi Complete viscometer (Staufen, Germany) with an accuracy of 1% and a repeatability of 0.2%. The tested liquid was placed in a measuring vessel with an appropriate spindle, and the measurement was recorded after a 10 min stabilization period. Each viscosity measurement was performed three times at the specified temperature. The results of the experiments are presented in Section 3.1.

Tribological tests were conducted on a TRB^3^ tribometer (Anton Paar, Baden, Switzerland) using a ball-on-disc configuration. The tribometer has a friction force resolution of 0.06 mN (or 0.015 mN optionally). The tests were performed under the following conditions:

- Load (P) = 10 N;
- Sliding velocity (v) = 0.1 m/s;
- Sliding distance (s) = 1000 m;
- Friction node: a ball of 100Cr6 steel—a disc of 100Cr6 steel;
- Radius: 8 mm;
- Lubricant—ionic liquid:
  ◦ Tributyl(methyl)phosphonium dimethyl phosphate 97%—MFCD;
  ◦ 1-Butyl-3-methylimidazolium hexafluorophosphate 97%—BMIMPF6;
  ◦ 1-Butyl-3-methylimidazolium tetrafluoroborate 98%—BMIMBF4;
- The amount of the ionic liquids during tests at ambient temperature and 40 °C: about 3 mL and 40 mL, respectively;
- Test execution temperatures: ambient (25 ± 1.5 °C) and 40 °C;
- Humidity: 40 ± 0.5%.

After the tribological tests, the surface geometry of the discs and balls was examined using a Leica DCM8 confocal microscope (Leica Geneva, Switzerland) in interferometric mode. The microscope has an accuracy of <3% relative error (open loop) and <20 nm error (closed loop) at 20× magnification. The results of this analysis are presented in Section 3.2. The results of the experiments are presented in Section 3.2.

**Figure 1:**
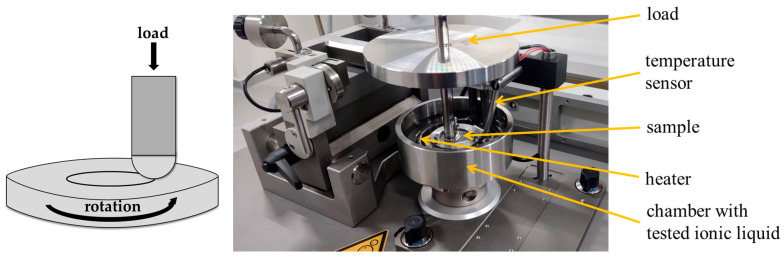
TRB^3^ tribometer: diagram and view of friction node.

The results of the tribological tests are presented in Section 3.3. Figure 1 presents a schematic of the friction node and a view of the tester during operation.

Discs and balls made of 100Cr6 steel were used for the tribological tests. 100Cr6 steel is a high-carbon steel commonly used for ball and rolling bearings. Its chemical composition is summarized in Table 3 [35].

The friction coefficient and linear wear of the contacting surfaces were determined during tribological tests. Subsequently, the wear tracks on the discs and balls were examined using the Leica DCM8 confocal microscope (Leica Geneva, Switzerland) in interferometric mode. The results of the experiments are presented in Section 3.4.

The chemical composition of the samples and counter-samples was analyzed using a Phenom XL scanning electron microscope equipped with an EDS microanalyzer (PhenomWorld, Eindhoven, Netherlands). The microscope was operated at an accelerating voltage of 5–15 kV. The results of the experiments are presented in Section 3.5.

## 3. Results

### 3.1. Density and Dynamic Viscosity Results

**Figure 2:**
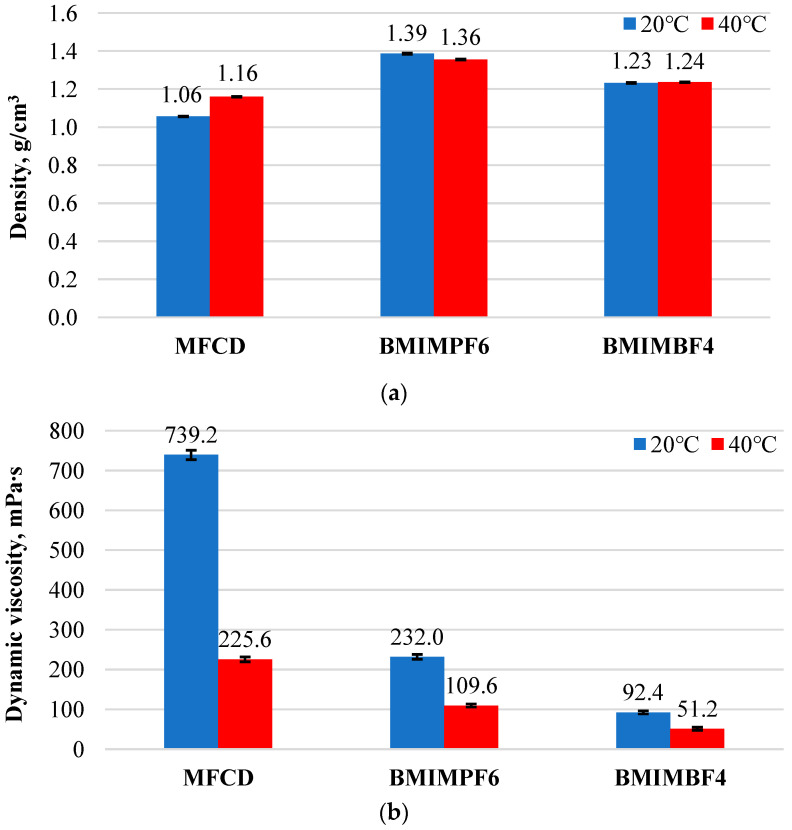
Test results: (**a**) density and (**b**) dynamic viscosity of tested ionic liquids at 20 °C and 40 °C.

Figure 2 shows the mean density values (Figure 2a) and dynamic viscosity (Figure 2b), including standard deviation.

The density and dynamic viscosity of the ionic liquids varied with temperature. As the temperature increased, the dynamic viscosity of all tested ionic liquids decreased (Figure 2b). This decrease was particularly pronounced for MFCD, where its viscosity was reduced by more than threefold. In contrast, the viscosity of BMIMPF6 and BMIMBF4 decreased by approximately half. Regarding density (Figure 2a), the density of MFCD increased slightly with temperature. However, the densities of BMIMPF6 and BMIMBF4 remained relatively constant across the temperature range.

### 3.2. Confocal Microscopy Results

**Figure 3:**
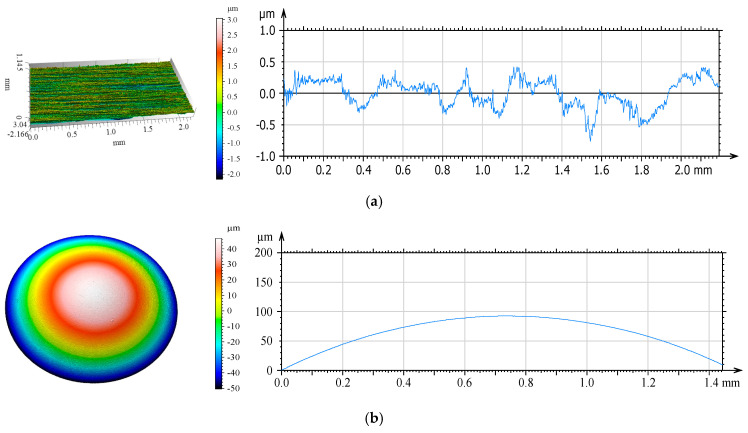
Isometric views and primary profiles of steel samples: (**a**) disc; (**b**) ball.

Figure 3 shows the isometric images and surface profiles of the discs and balls used in the tribological tests.

The geometric structure of the disc and ball surfaces was analyzed. The initial analysis of the primary profiles revealed numerous irregularities on the steel disc surface (Figure 3a). These irregularities included elevations of approximately 0.5 µm and depressions of approximately 0.7 µm.

### 3.3. Tribological Tests

**Figure 4:**
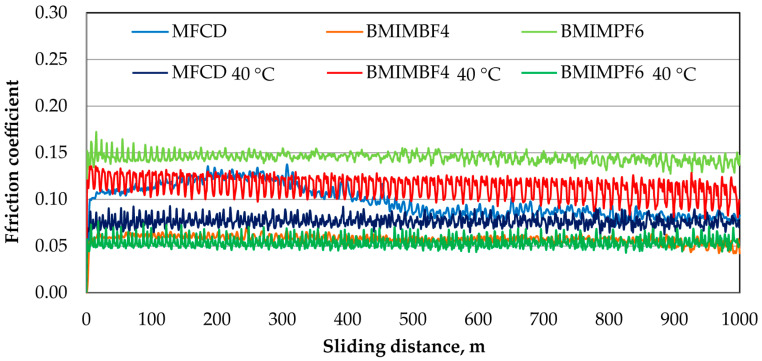
Plots of the friction coefficients as a function of sliding distance at ambient temperature and 40 °C for the ionic liquids under test.

**Figure 5:**
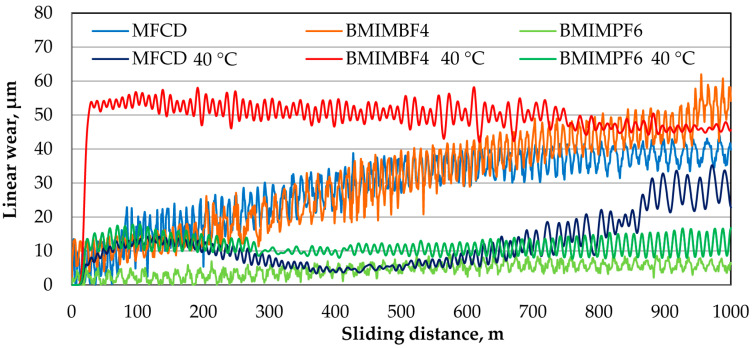
Plots of linear wear as a function of sliding distance at ambient temperature and 40 °C for the ionic liquids under test.

Figure 4 and Figure 5 present the friction coefficient and linear wear as functions of sliding distance at ambient temperature and 40 °C, respectively.

Figure 4 and Figure 5 illustrate the friction coefficient and linear wear as functions of sliding distance at ambient temperature and 40 °C, respectively.

At ambient temperature, BMIMBF4 provided the lowest friction coefficient (Figure 4), maintaining high stability throughout the test. Conversely, BMIMPF6 showed the highest friction coefficient. At 40 °C, BMIMPF6 demonstrated the lowest friction coefficient, while BMIMBF4 exhibited the highest.

At ambient temperature, BMIMPF6 exhibited the lowest linear wear (Figure 5), while BMIMBF4 showed the highest. MFCD initially displayed a similar wear level to BMIMPF4 but experienced a decrease toward the end of the test. At 40 °C, BMIMPF6 again demonstrated the lowest linear wear and BMIMBF4 the highest. In a specific test range, MFCD initially provided lower wear than BMIMPF6 but eventually increased, although still remaining much lower than that of BMIMBF4.

At 40 °C, BMIMPF6 provided the lowest friction coefficient and linear wear, while BMIMBF4 showed the highest. This trend was not observed at ambient temperature. The superior performance of BMIMPF6 at elevated temperatures can be attributed to its higher density and viscosity, which enhance its ability to adhere to surfaces, fill irregularities, and provide effective lubrication and separation of the contacting surfaces.

### 3.4. Confocal Microscopy Results After Tribological Tests

**Figure 6:**
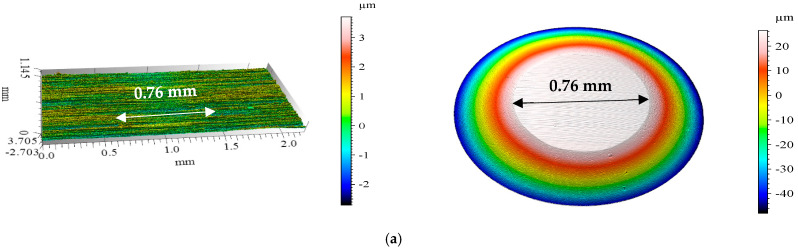

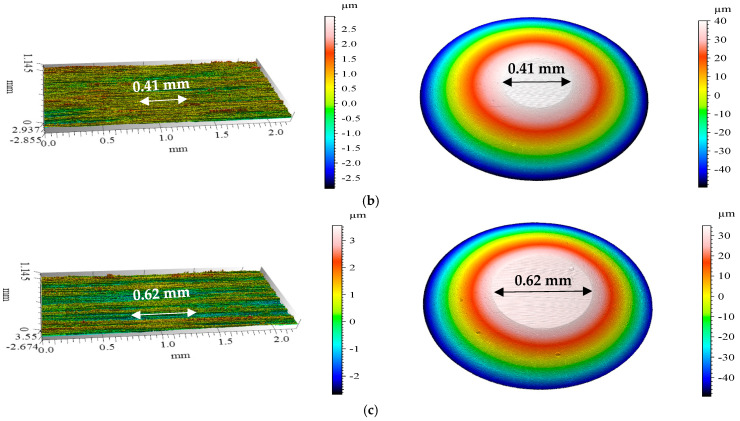
Isometric views of the discs and balls after the tribological tests at ambient temperature for the ionic liquids under test: (**a**) MFCD, (**b**) BMIMPF6, and (**c**) BMIMBF4.

Figure 6 presents the isometric images and primary steel disc and ball profiles after wear tests under ionic liquid lubrication conditions at ambient temperature.

After the tribological tests, surface geometry was examined. At ambient temperature, BMIMPF6 provided the least wear, with minimal wear tracks on both the disc and ball (Figure 6b). A closer examination (Table 3) revealed that the track depths were within the sample’s roughness profile, which was easily discerned. In contrast, MFCD lubrication led to the most significant wear (Figure 6a), as confirmed by the largest wear area (Table 4) and high linear wear values.

**Figure 7:**
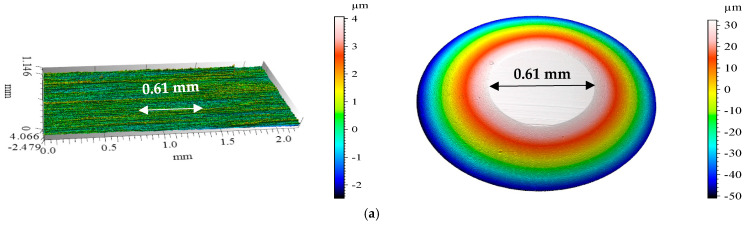

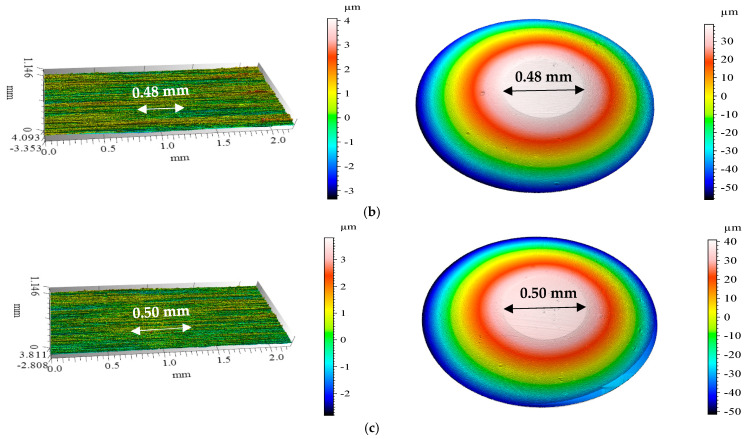
Isometric views of the discs and balls after the tribological tests at 40 °C for the ionic liquids under test: (**a**) MFCD, (**b**) BMIMPF6, and (**c**) BMIMBF4.

Figure 7 presents the isometric images and primary profiles of the steel samples and counter-samples after testing at 40 °C. Table 4 summarizes the maximum depth, height, and area of wear features on the discs at both ambient temperature and 40 °C.

Wear analysis at 40 °C shows that with BMIMBF6, both the sample and counter-sample exhibited negligible wear (Figure 7b). This finding is supported by the lowest linear wear value compared to the other ionic liquids. In contrast, the use of MFCD resulted in the highest wear on the disc and ball. The low density of MFCD may have hindered the formation of an effective lubricating film, leading to increased friction and wear.

Table 5 and Table 6 present the surface roughness parameters of the discs and balls before and after the tribological tests at ambient temperature and 40 °C, respectively.

The surface roughness analysis conducted after the tests at ambient temperature (Table 5) showed that BMIMPF6 lubrication resulted in the lowest surface roughness values for both the disc and ball (Table 5), indicating a smoother surface compared to the other lubricants. However, this trend was not observed at 40 °C (Table 6). Interestingly, BMIMPF6 consistently yielded the lowest Sv parameter values at both temperatures, suggesting a smoother surface with fewer pits (valleys) in the wear track.

Equations (1) and (2) were used to calculate ball wear after the tribological tests with ionic liquid lubrication. The results of ball wear are shown in Table 7.

(1) $$V_{ball}=\frac{1}{3}\pi h^{2}\left(3R-h\right)$$

(2) $$h=R-\sqrt{R^{2}-r^{2}}$$

where:

- r—the radius of the wear track (mm);
- h—the height of the worn surface (mm);
- R—the radius of the sphere (mm).

As the temperature increased, ball wear decreased for MFCD and BMIMBF4. However, BMIMPF6 showed a slight increase in wear with increasing temperature. At ambient temperature, BMIMPF6 provided the lowest ball wear, while MFCD showed the highest. At 40 °C, BMIMPF6 again achieved the lowest wear, and MFCD the highest. The most significant impact of temperature was observed for MFCD, where wear increased substantially at higher temperatures.

### 3.5. Scanning Electron Microscopy Results

**Figure 8:**
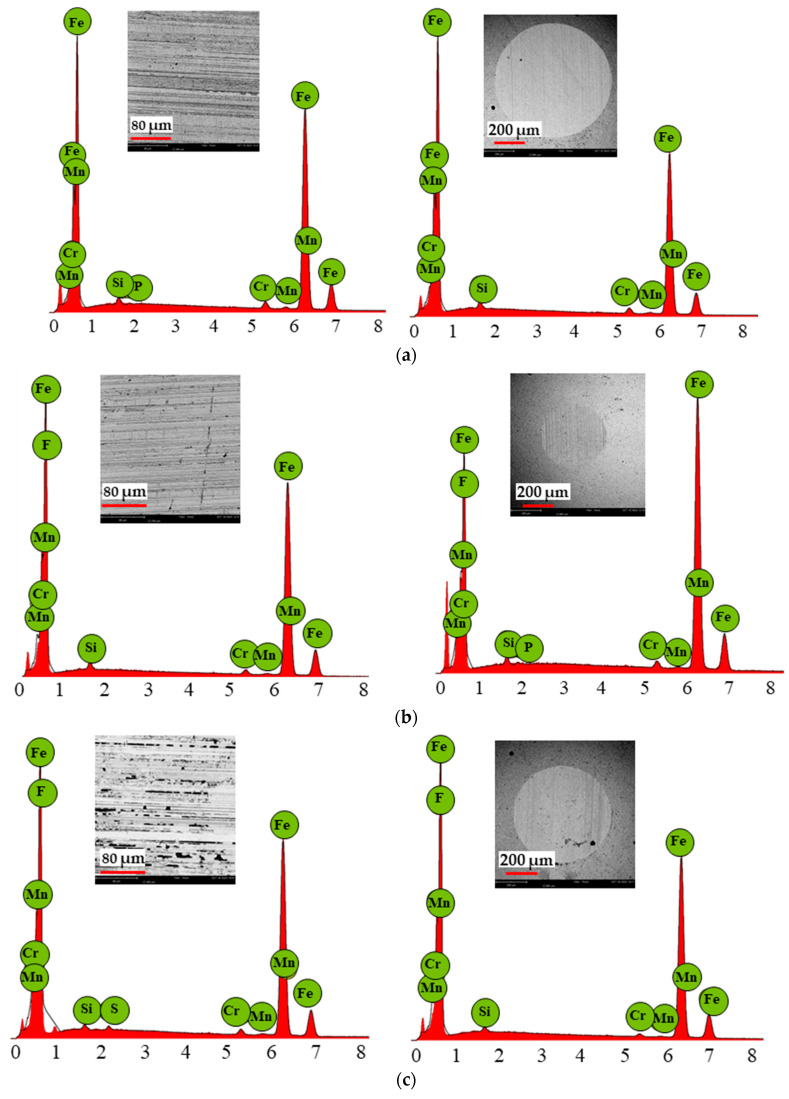
EDS patterns of wear tracks resulting from sliding contact on discs and balls after tribological test with tested ionic liquids at ambient temperature: (a) MFCD, (b) BMIMPF6, and (c) BMIMBF4.

**Figure 9:**
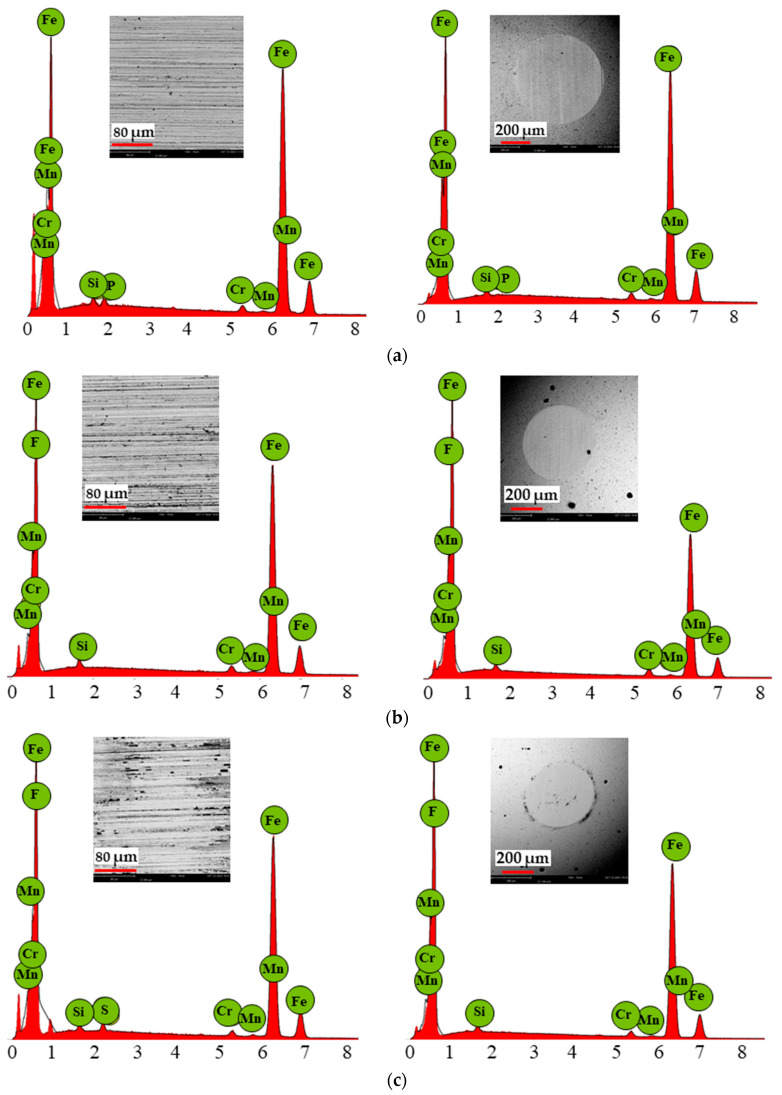
EDS patterns of wear tracks resulting from sliding contact on discs and balls after tribological test with tested ionic liquids at 40 °C: (**a**) MFCD, (**b**) BMIMPF6, and (**c**) BMIMBF4.

Figure 8 and Figure 9 show the point analysis and morphology of the steel discs and balls after the tribological tests under lubrication with the ionic liquids at ambient temperature and 40 °C. Table 8 and Table 9 present the averaged chemical composition from the wear track at the selected point.

The EDS analysis of the wear tracks on the discs and balls after the tribological tests with BMIMPF6 and BMIMBF4 at ambient temperature and 40 °C (Table 7 and Table 8) revealed the presence of fluorine on both surfaces. At ambient temperature, higher fluorine concentrations were observed on the disc and ball lubricated with BMIMBF4. However, at 40 °C, BMIMPF6 exhibited the highest fluorine concentration. Additionally, phosphorus was detected on the ball after BMIMPF4 lubrication at ambient temperature and on the disc at 40 °C.

During friction, iron fluorides are formed on frictionally cooperating metal surfaces, which help to improve tribological properties. However, their disadvantage from a chemical point of view is the hydrolysis of iron fluorides. This reaction results in the formation of iron hydroxides and iron oxides, causing iron rusting. Iron fluorides are one of the Lewis acids that catalyze lubricant degradation [36]. In addition to fluorine, phosphorus was also observed after friction with BMIMPF6 ionic liquid lubrication. The phosphate-type anion is reactive and controls the thermal stability and degradation of the ionic liquid, resulting in lower wear. An increase in temperature increases the rate of thermal degradation. Lubricant degradation starts with the anion [37,38].

Additionally, Forbes et al. [39,40] investigated the tribological properties of dialkyl phosphates, finding that short-chain esters exhibit good extreme pressure (EP) properties, while long-chain esters provide better anti-wear (AW) properties. Sakurai et al. [41] also studied phosphate-based additives and observed a correlation between wear reduction and reactivity with metal surfaces under friction. Their tribofilm analysis found that dialkyl phosphates formed alkaline iron phosphate films on frictionally cooperating metal surfaces [42]. These findings suggest that phosphorus-containing additives can form protective films on metal surfaces, reducing friction and wear.

The confocal microscope and SEM images clearly show pitting on both the disc and the ball after the tribological tests with BMIMPF6 and BMIMBF4 liquid lubrication at ambient temperature and 40 °C. These were created by the ionic liquid consisting of cations and anions used for tribological testing. Anions have a significant effect on the tribological properties of ionic liquids. Hydrophobic anions, such as BF4 and PF6, sometimes cause steel corrosion. PF6 decomposes via hydrolysis to form hydrogen fluoride. However, other hydrophobic anions that are less corrosive show good tribological properties [43]. According to Wang et al. [44], cations of ionic liquids contribute to corrosion on material surfaces. In addition, they observed that copper, carbon steel, and magnesium exhibit little corrosion resistance when in contact with some ILs. However, the most visible corrosion on the steel occurs in contact with the ionic liquid BMIMBF4, as seen in the images (Figure 8c and Figure 9c). The observed corrosion, particularly with BMIMBF4, is likely exacerbated by water in the ionic liquid, as confirmed by other studies [37,45]. Kinoshita et al. [46] conducted tribological tests with ionic liquid (BMIMBF4) lubrication without and with 0.2, 2.0, and 10.0 mass % graphene oxide. They demonstrated that the corrosion formed on the sample surface lubricated with pure ionic liquid could be caused by the formation of metal fluoride on the frictionally interacting surfaces (tribological system, friction node) as a result of tribochemical reactions (of the steel sample with the ionic liquid). The resulting corrosion would have indicated a robust tribochemical reaction when lubricated with pure ionic liquid. However, only a small amount of evident corrosion was observed when lubricated with mixtures of ionic liquid and graphene oxide. Despite the formation of corrosion, the tribofilm consisting of fluorine reduced the friction coefficient. Li et al. [8] demonstrated that graphene oxide in water as a coolant also serves as an excellent lubricant, capable of significantly reducing the friction coefficient, for example, during grinding processes.

Analysis of the results obtained in the present study showed a similarity with the results reported by Kinoshita et al. [46]. The tribofilm formed, consisting of fluorine, contributed to the lowest friction coefficient value at ambient temperature. However, pitting was also observed at the wear track, indicating the formation of corrosion at both ambient temperature and 40 °C. According to Zhang et al. [47], the ionic liquid containing phosphate and imidazole ions showed very good lubricating and anti-wear properties and did not cause corrosion on the metal surface. Similarly, Minami [43] observed superior tribological properties and thermal stability for BMIMPF6 compared to BMIMBF4.

## 4. Conclusions

The aim of this study was to compare the tribological and rheological properties of selected ionic liquids. The following conclusions were drawn from the conducted research:

1. Temperature was found to influence the density and viscosity of the ionic liquids. The density behavior varied, while the dynamic viscosity of all ionic liquids decreased with increasing temperature. BMIMPF6 showed a decrease in density, whereas MFCD and BMIMBF4 exhibited an increase in density as temperature increased.
2. After conducting tribological tests under ambient conditions, the BMIMBF4 ionic liquid exhibited the lowest coefficient of friction. On the other hand, the BMIMPF6 ionic liquid achieved the lowest linear wear, which is supported by the minimal wear traces observed on the disc and ball.
3. Tests of friction at 40 °C showed that the BMIMPF6 ionic liquid gave the least friction and wear. This is because the fluorine in the liquid helped create a protective layer that reduced wear, and the liquid itself was dense and viscous enough to fill in gaps on the surfaces, providing good lubrication.
4. The increased temperature contributed to a higher phosphorus concentration on both the disc and ball surfaces during lubrication with the MFCD ionic liquid. A tribofilm formed, acting as an anti-wear surface layer, which reduced wear on both the sample and counter-sample.
5. A fluorine layer formed on the discs and balls during the tribological tests with BMIMBF4 and BMIMPF6 ionic liquids and caused corrosion at room temperature and 40 °C. Corrosion was less severe when both fluorine and phosphorus (in BMIMPF6) were present. Conversely, corrosion was more pronounced when only BF4- anions (from BMIMBF4) were present. Pitting was observed on the surfaces of the discs and balls.

## Figures and Tables

**Table 1:** The composition and information of the constituents of the ionic liquids [33,34].

Short Name	Chemical Formula	Chemical Structure	Molecular Weight, g/mol
MFCD	C_15_H_36_O_4_P_2_	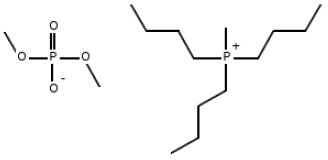	342.39
BMIMPF6	C_8_H_15_F_6_N_2_P	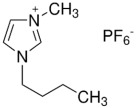	284.18
BMIMBF4	C_8_H_15_BF_4_N_2_	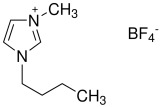	226.02

**Table 2:** Basic parameters of ionic liquids [33,34].

Short Name	Purity, %	Amount of Water, %	Color	State of Concentration
MFCD	97	X	colorless to light yellow	liquid
BMIMPF6	≥97.0 (HPLC)	X	light yellow	liquid
BMIMBF4	≥98	≤0.5%	yellow	liquid

**Table 3:** Chemical composition of 100Cr6 steel [35].

Element	C	Cr	Mn	Mo	Ni	P	S	Si	Al	Cu
Weight, %	0.95–1.10	1.35–1.60	0.25–0.45	max 0.10	max 0.30	max 0.03	max 0.02	0.15–0.35	max 0.05	max 0.35

**Table 4:** Disc wear after the tribological tests under lubrication with the tested ionic liquids at ambient temperature and 40 °C.

Temperature	Ionic Liquid	Max. Depth, µm	Wear Track Area, µm^2^	Max. Height, µm	Peak Area, µm^2^
Ambient temperature	MFCD	0.40	87.59	0.37	47.6
	BMIMPF6	0.18	3.65	0.41	54.94
	BMIMBF4	0.60	60.12	0.50	69.39
40 °C	MFCD	0.37	52.03	0.37	55.21
	BMIMPF6	0.71	125.40	0.33	9.18
	BMIMBF4	0.30	19.76	0.54	68.00

**Table 5:** Surface roughness parameters for the discs and balls before and after the tribological tests at ambient temperature.

Surface Roughness Parameters		Reference		MFCD		BMIMPF6		BMIMPF4	
		Disc	Ball	Disc	Ball	Disc	Ball	Disc	Ball
Sq	µm	0.42	0.23	0.78	3.83	0.46	1.34	0.89	3.01
Ssk	-	−0.31	0.70	−0.11	−0.57	−0.66	−2.22	−0.12	−1.10
Sku	-	3.16	3.86	2.63	2.33	3.72	7.79	3.11	3.45
Sp	µm	2.54	3.38	2.34	6.78	2.19	2.96	2.60	4.58
Sv	µm	1.81	1.29	2.89	8.74	2.16	5.54	2.99	8.53
Sa	µm	0.33	0.18	0.63	3.15	0.36	0.87	0.70	2.32

**Table 6:** Surface roughness parameters for the discs and balls before and after the tribological tests at 40 °C.

Surface Roughness Parameters		Reference		MFCD		BMIMPF6		BMIMPF4	
		Disc	Ball	Disc	Ball	Disc	Ball	Disc	Ball
Sq	µm	0.42	0.23	0.79	3.08	0.76	1.79	0.51	1.78
Ssk	-	−0.31	0.70	−0.31	−1.07	0.00	−1.82	−1.03	−1.62
Sku	-	3.16	3.86	2.79	3.39	2.93	5.85	4.75	5.11
Sp	µm	2.54	3.38	2.51	6.09	2.82	4.37	3.53	4.09
Sv	µm	1.81	1.29	3.04	8.60	2.51	6.41	2.82	6.99
Sa	µm	0.33	0.18	0.64	2.39	0.61	1.25	0.39	1.28

**Table 7:** The wear of the balls after the tribological tests under lubrication with the tested ionic liquids at ambient temperature and 40 °C.

Temperature	Ionic Liquid	Wear of the Balls		
		Diameter, mm	High h, mm	Volume V, mm^3^
Ambient temperature	MFCD	0.76	0.048	0.044
	BMIMPF6	0.41	0.014	0.004
	BMIMBF4	0.62	0.032	0.019
40 °C	MFCD	0.61	0.031	0.018
	BMIMPF6	0.48	0.019	0.007
	BMIMBF4	0.50	0.021	0.008

**Table 8:** Elements wt. % present on discs and balls after tribological tests at ambient temperature.

Ionic Liquid	MFCD		BMIMPF6		BMIMBF4	
Element	Disc	Ball	Disc	Ball	Disc	Ball
Fe	97.51	97.51	85.74	87.60	85.09	85.74
F	-	-	12.54	10.62	13.34	12.54
Cr	1.27	1.42	1.05	0.79	1.02	1.05
Si	0.88	0.85	0.52	0.51	0.36	0.52
Mn	0.25	0.17	0.15	0.26	0.19	0.15
P	-	-	-	0.23	-	-
S	-	-	-	-	-	-

**Table 9:** Elements wt. % present on discs and balls after tribological tests with tested ionic liquids at 40 °C.

Ionic Liquid	MFCD		BMIMPF6		BMIMBF4	
Element	Disc	Ball	Disc	Ball	Disc	Ball
Fe	97.76	97.83	85.61	81.76	86.08	84.09
F	-	-	12.28	16.25	11.23	13.75
Cr	1.23	1.20	1.14	1.32	0.94	1.16
Si	0.73	0.63	0.62	0.48	0.54	0.49
Mn	0.26	0.22	0.21	0.19	0.22	0.25
P	-	-	0.15	-	-	-
S	-	-	-	-	0.99	-

## Data Availability

The original contributions presented in this study are included in the article; further inquiries can be directed to the corresponding author.
